# Supplementary material for: Development of a Forced-Choice Personality Inventory via Thurstonian Item Response Theory (TIRT)
Source: Behav Sci (Basel). 2024 Nov 21;14(12):1118. doi: 10.3390/bs14121118 (PMC11673971; doi:10.3390/bs14121118)
Supplement: Supplementary file 1 [file behavsci-14-01118-s001.zip › behavsci-3199525-supplementary.pdf]

Table S1

Factor Loadings of the Final Item Pool for the Extraversion Factor

Items	Factor Loadings
**EX12**	.80
**EX 13**	.76
**EX 11**	.73
**EX 27**	.69
**EX 21**	.66
**EX 23**	.66
**EX 26**	.59
**EX 34**	.59
**EX 8**	.57
**EX 22**	.56
**EX 7**	.55
**EX 40**	.54
**EX 35**	.45
**EX 5**	.43
**EX 28**	.34

Table S2

Factor Loadings of the Final Item Pool for the Emotional Stability Factor

Items	Factor Loadings
**ES5**	.68
**ES9**	.63
**ES40**	.59
**ES30**	.59
**ES14**	.58
**ES15**	.55
**ES19**	.53
**ES20**	.51
**ES22**	.50
**ES34**	.50
**ES24**	.44
**ES39**	.42
**ES25**	.39
**ES35**	.38
**ES21**	.36

Table S3

Factor Loadings of the Final Item Pool for the Openness to Experience Factor

Items	Factor Loadings
**O3**	.67
**O28**	.67
**O23**	.67
**O27**	.66
**O34**	.66
**O17**	.64
**O40**	.61
**O2**	.60
**O1**	.59
**O8**	.58
**O6**	.53
**O22**	.50
**O35**	.49
**O39**	.45
**O11**	.45

Table S4

Factor Loadings of the Final Item Pool for the Agreeableness Factor

Items	Factor Loadings
**A26**	.77
**A18**	.76
**A8**	.72
**A33**	.67
**A21**	.66
**A35**	.64
**A27**	.63
**A14**	.62
**A7**	.62
**A15**	.60
**A16**	.59
**A4**	.58
**A39**	.55
**A28**	.52
**A40**	.50

Table S5

Factor Loadings of the Final Item Pool for the Conscientiousness Factor

Items	Factor Loadings
**C15**	.68
**C32**	.67
**C30**	.64
**C2**	.63
**C31**	.62
**C33**	.60
**C3**	.58
**C38**	.57
**C36**	.57
**C9**	.55
**C21**	.53
**C29**	.53
**C10**	.53
**C14**	.51
**C1**	.51
